# Perspectives on the Mature T-Cell Lymphomas in the Middle East: A Comprehensive Review of the Present Status

**DOI:** 10.3390/cancers16244131

**Published:** 2024-12-11

**Authors:** Mubarak Al-Mansour, Syed Sameer Aga, Owen A. O’Connor

**Affiliations:** 1 Adult Medical Oncology, Princess Noorah Oncology Centre, Ministry of National Guard Health Affairs-Western Region (MNGHA-WR), King Saud Bin Abdul Aziz University for Health Sciences (KSAU-HS), King Abdullah International Medical Research Centre (KAIMRC), King Abdulaziz Medical City, Jeddah 21423, Saudi Arabia; mansourmu@ksau-hs.edu.sa; 2 College of Medicine, King Abdullah International Medical Research Centre (KAIMRC), King Saud Bin Abdul Aziz University for Health Sciences (KSAU-HS), Ministry of National Guard Health Affairs-Western Region (MNGHA-WR), King Abdulaziz Medical City, Jeddah 21423, Saudi Arabia; 3 University of Virginia Comprehensive Cancer Center, Translational Orphan Blood Cancer Research Center, Charlottesville, VA 22903, USA; oao2j@uvahealth.org

**Keywords:** peripheral T-cell lymphoma, anaplastic large-cell lymphoma, cutaneous T-cell lymphoma, angioimmunoblastic T-cell lymphoma, adult T-cell leukemia/lymphoma, Middle East

## Abstract

**Highlights:**

The prevalence of TCLs in the ME is not well documented, as most of the research has been conducted in Western countries. While recent studies suggest that the prevalence of TCLs in the ME is comparable to that of Western countries, there is a suggestion that the prevalence of T-cell lymphomas may be increasing in the ME compared to the previous decade. Because of the limited data available on TCLs in the ME, its epidemiology is not well understood. However, there are some studies that demonstrated that the risk of TCLs is significantly higher in people who have a family history of lymphoma, those who have a history of autoimmune diseases such as rheumatoid arthritis and systemic lupus erythematosus, and those who have a history of exposure to ionizing radiation or certain chemicals such as benzene.

**Why are the main findings?**

■ Diagnosis of TCLs in the ME is often complicated and requires pathologists familiar with the complex classification, specialized techniques in immunohistochemistry and flow cytometry, and imaging, including PET scans. The use of molecular testing and profiling of TCLs in the region is limited.
■ The treatment of TCLs in the ME, similar to other parts of the world, is often confusing and lacks consensus. Treatments may include combination chemotherapy, radiation therapy, immunotherapy, and autologous or allogeneic stem cell transplant. Chemotherapy is the most common treatment option for TCLs in the ME and is usually used in combination with other modalities, such as radiation therapy or immunotherapy.

**What is the Implications of main findings?**

■ The clinical outcomes of TCLs in the ME are not well understood, as there is limited data available on the outcomes of these cancers in the region. However, some studies have looked at the outcomes of T-cell lymphomas in the ME, suggesting that the 5-year survival rate for patients with TCLs is comparable to that of patients in Western countries.
■ There is a dearth of real-world evidence and management consensus in the region, as well as a restricted number of clinical trials resulting from the ME pertaining to the management of TCLs. One of the main reasons for the limited evidence arising from the region is the lack of TCL registries.

**Simple Summary:**

This review study focusses on T-cell lymphomas (TCLs), a type of rare and severe disease that is prevalent in the Middle East. We attempted to highlight the fact that there is less data on the occurrence, treatment, and results of TCLs in this region than in Western countries. The purpose of this research was to highlight the need for further clinical trials and registries to better understand these diseases and enhance patient care. To improve diagnosis and treatment strategies, we recommend centralizing laboratories and establishing a unified registry. Overall, the study intends to increase awareness of TCLs in ME and advocate for better research and healthcare practices to enhance patient outcomes.

**Abstract:**

**Background:** T-cell lymphomas (TCLs) are rare and aggressive malignancies associated with poor outcomes, often because of the development of acquired drug resistance as well as intolerance to the established and often toxic chemotherapy regimens in elderly and frail patients. The many subtypes of TCL are well established to exhibit marked geographic variation. The epidemiology, clinical presentation, diagnosis, prognosis, and treatment of TCLs in the Middle East (ME) are yet to be explored; hence, limited data are available about these entities in this part of the world. **Aim**: Therefore, in this review article, we aim to discuss the available data regarding the T-cell neoplasms in the ME, including the incidence of specific subtypes of peripheral T-cell lymphoma (PTCL), as well as the trends in survival and treatment, all in an effort to understand the natural history of these complex entities across the ME.

## 1. Introduction

T-cell lymphomas (TCLs), which account for approximately 12% of all NHL, are a heterogeneous group of rare malignancies that range from relatively indolent to highly aggressive [1]. Cancers of the NK cell are also included in this category because of their close relationship to T-cell malignancies. The frequency of different TCL subtypes varies by region and type. They can be common TCLs like (a) peripheral T-cell lymphoma not otherwise specified (PTCL-NOS), which is the most common subtype, accounting for about 30% of all T-cell lymphomas; (b) angioimmunoblastic T-cell lymphoma (AITL), which represents about 15–20% of all T-cell lymphomas and is more common in older adults; or (c) anaplastic large cell lymphoma (ALCL), accounting for about 12–15% of all T-cell lymphomas and is more common in children and young adults. While on the other side, they can be rare, like (a) adult T-cell leukemia/lymphoma (ATLL), which is rare outside HTLV-1 endemic areas such as Japan, the Caribbean, and parts of central Africa; enteropathy-associated T-cell lymphoma (EATL), a rare subtype associated with celiac disease; or (b) hepatosplenic T-cell lymphoma (HSTL), extremely rare and aggressive subtype [1,2,3]. Adult T-cell leukemia/lymphoma (ATL) is rare in the Middle East but more common in Japan, the Caribbean, and parts of South America due to the higher prevalence of HTLV-1 infection. The incidence of ATL varies from 0.05 per 100,000 in the USA to as high as 27 per 100,000 in Japan [1,3]. The World Health Organization (WHO) published a new classification for lymphomas, separating T-cell/NK-cell lymphomas into precursor and mature T-cell neoplasms and defining four subtypes of mature lymphomas: cutaneous, nodal, extranodal, and leukemic [2]. TCLs can also be classified as indolent or aggressive lymphomas, with the majority of subtypes exhibiting an aggressive natural history. While indolent diseases like mycosis fungoides (MF), for example, can be characterized by a prolonged disease course managed with single agent or topical therapies, the aggressive diseases often have an acute presentation with B symptoms and rapid disease progression, and typically require combination chemotherapy. Entities like peripheral T-cell lymphoma not otherwise specified (PTCL-NOS), angio-immunoblastic T-cell lymphoma, anaplastic large T-cell lymphoma, and HTLV-1 associated adult T-cell leukemia and lymphoma are all regarded as highly aggressive diseases, MF and large granular T- or NK cell leukemia are generally regarded as more indolent diseases [3]. Herein, we aim to comprehensively summarize the current evidence regarding the regional features of the PTCL, including their epidemiology, clinical presentation, prognosis, and treatment of these types.

## 2. Peripheral T-Cell Lymphoma (PTCL)

### 2.1. PTCL, Not Otherwise Specified (PTCL-NOS)

Peripheral T-cell lymphomas (PTCLs) are a rare and heterogeneous group of lymphoid malignancy that results from the malignant transformation of mature, post-thymic (“peripheral”) T cells. The disorders are classified into leukemic, extranodal, cutaneous, and nodal forms [2]. However, according to the recent International Consensus Classification (ICC) report, primary nodal EBV-positive T- or NK-cell lymphoma is considered a provisional entity [4]. PTCL not otherwise specified (PTCL-NOS) refers to those entities that cannot be further classified into other subtypes [5,6,7]. Therefore, it is critical to establish a precise characterization of this heterogeneous group of patients.

#### 2.1.1. Epidemiology

According to several worldwide registries, PTCL-NOS is the most common subtype of PTCL worldwide, with a reported incidence of 22–36% [7]. It accounts for 25–36% of PTCL cases in North America and Europe. While PTCL-NOS was the second most frequent TCL in Asia, accounting for 22% of cases, adult T-cell lymphoma/leukemia (ATLL) was the most prevalent, accounting for about 25% of cases [8,9,10]. Blacks had a higher incidence of PTCL-NOS with an incidence rate (IR) of 1.67 (95% CI: 1.53–1.82) per 100,000 persons, in comparison with Alaska Natives, American Indians, Pacific Islanders, Asians, Hispanics, and non-Hispanic whites [11]. Worldwide, the disease exhibits a male preponderance of roughly 1.9:1, and the median age at presentation is about 60 [12,13]. The most commonly cited risk factors for the disease include a family history of hematologic malignancies, a smoking history of 40 years or more, psoriasis, and Celiac disease. A lower risk of the disease has been associated with alcohol consumption at least once a month, spending time in the sun, and a history of allergies [14].

In the ME, there is a paucity of data on the epidemiology of PTCL-NOS. Only a few scattered studies have been published to date, and these largely focused on the overall prevalence of PTCL and not the epidemiology of specific subtypes. Perry et al. reported that the overall prevalence of PTCL was 6.1% in Algeria, 3.8% in Egypt, 8.1% in Saudi Arabia, 7.7% in Kuwait, and 7.3% in the rest of the Middle East/North Africa (MENA) region [15]. The highest prevalence of PTCL was observed in Lebanon (18.4%) [16], followed by Saudi Arabia (11.3%) [17], Jordan (10.8%) [18], UAE (9%) [19], Turkey (7.7%) [20], Iraq (6.8%) [21], Egypt (5.8%) [22,23], Kuwait (5.2%) [24], and Iran (1.9%) [25]. Even though there is a wide range in incidence shown by these figures, some of the variations may be linked to regional variations in the experience of the diagnostic pathologists as well as variations in the quality of the databases used.

The most common subtype in Iran was PTCL-NOS, with a prevalence of 3.9% among all cases and 44.8% among TCL cases [26]. With an overall incidence of 20.3%, PTCL-NOS was found to be the most common TCL in an Algerian registry. Additionally, Caucasians had a higher prevalence of PTCL-NOS (64.4% vs. 30.5% vs. 5.1%) than Blacks or North Africans [27]. Approximately 13% of all TCLs and 2.3% of all cases in Turkey were considered to be PTCL-NOS [28].

#### 2.1.2. Clinical Presentation

Across the worldwide registries, the median age of patients with PTCL-NOS is 60 years, exhibiting a slight male preponderance (M:F ratio 1.5–1.9 to 1.0) [13,29,30]. Often, bulky nodal disease is the most common clinical presentation. Approximately 69% are diagnosed with advanced stages (III/IV), and 50–70% have felt to have high to high-intermediate scores on the International Prognostic Index (IPI) [10,13]. Although extranodal disease may involve any site and commonly coexists with nodal disease (49%), it typically affects the lungs, bone marrow, skin, and gastrointestinal tract [10,13,29,31,32]. Up to 35% of patients have B symptoms, which are associated with hemophagocytic syndrome (HPS) [13,31,32,33].

Compared to western nations, the ME has a larger male preponderance and a somewhat lower mean age at diagnosis. The male-to-female ratio among patients with PTCL-NOS was found to be 4.2:1 in Iran [26], 3:1 in Saudi Arabia [17], 3:1 in Bahrain [34], 2.4:1 in Jordan [35], 2:1 in the UAE [19], 1.7–2:1 in Iraq [21,36], and 1.4:1 in Algeria [27]. Similarly, the mean age at diagnosis was also found to vary by ME region, being 54.1 ± 15.5 in Iran [26], 55.1 ± 13.7 in Algeria [27], 58 in Saudi Arabia [17], 53 in the UAE [19], 51 in Bahrain [34], 46 in Jordan [35], 40–45 in Iraq [21,36], and 35.5 in Egypt [23]. Similar to the global estimates, the nodal type of PTCL is more prevalent than extranodal in the ME. In Iran, among the patients with PTCL-NOS, the nodal-to-extranodal ratio was 1.8:1 [26]. In the Algerian population with PTCL, 45% were nodal, 21.7% leukemic, 15% cutaneous, 13.3% extranodal, and 3.4% lymphoblastic T lymphoma [27]. Among an Iraqi population with PTCL, there were no reported cases of extranodal involvement [36]. Similar to the global reports, the majority of the patients (81.4%) presented with advanced stages (III-IV) compared to early stages (18.7%) [27].

### 2.2. Angioimmunoblastic T-Cell Lymphoma (AITL)

Angioimmunoblastic T-cell lymphoma, also commonly referred to as AITL, is one of the more common subtypes of PTCLs characterized by hypergammaglobulinemia, anemia, hepatosplenomegaly, and generalized lymphadenopathy.

#### Epidemiology

AITL accounts for 15–20% of all PTCL cases, making it one of the most prevalent subtypes of T-cell lymphoma [10]. It represents around 28.7% of PTCL cases in Europe, which is higher than in Asia (17.9%) and North Africa (16.0%). The prevalence of AITL in Saudi Arabia was reported to be 10.86% among all patients with NHL, with a mean age at diagnosis of 62 years [17]. In Iran, the prevalence of AITL was 3.2% among all PTCL, with a mean age at diagnosis of 20 ± 0 years [26]; in the Iraqi PTCL patients, the prevalence was 1.04%, with a median age of 55 years and a predominance of males [36]; and in Bahrain, it was 6.25% and a mean age of 71 years [34].

### 2.3. Anaplastic Large-Cell Lymphoma (ALCL)

ALCL is a distinct subtype of PTCL, which is further categorized into three types, including (1) systemic anaplastic large cell lymphoma, sub-divided into AKL positive and ALK-negative; (2) cutaneous ALCL; and (3) breast implant-associated ALCL. Overall, the entity of ALCL is associated with a superior outcome compared to all other forms of PTCL. Historically, ALK = negative ALCL exhibited the worse prognosis of any subtype in the group; however, the introduction of Brentuximab vedotin into CHOP (Bv-CHP) has now shown that even this entity does equally as well as ALK-positive ALCL [37].

#### 2.3.1. Epidemiology

ALCL accounts for 10% to 15% of PTCLs, 10% to 20% of childhood lymphomas, and 3% to 5% of all NHLs. A comprehensive database employing population-based registry data indicated racial differences in ALCL incidence [11,38]. In the ME, the number of epidemiological studies regarding ALCL is very limited (Table 1). The overall prevalence ranged between 1.3% and 16.67%. The mean age of patients was comparable with the global patterns. Moreover, the majority of the studies showed a predominance of males [11,38].

#### 2.3.2. Extranodal NK/T Cell Lymphoma, Nasal Type (ENKTL, NT)

ENKTCL-NT is a rare form of NHL that primarily affects patients in Asian countries such as China, Japan, Taiwan, Thailand, Korea, Latin America, Mexico, Guatemala, Peru, and Brazil [41,42,43]. About 2.9% of NHL cases in Saudi Arabia were diagnosed with NKTCL [44,45]. Most affected patients are adults (46–60 years), though it can affect children as well. It is more common in males, proportionally representing 55–78% of cases worldwide [45,46,47].

## 3. Treatment Strategies

Frontline treatment for PTCL-NOS often consists of four to six cycles of combination chemotherapy, typically CHOP-based (cyclophosphamide, doxorubicin, vincristine, and prednisone) [13,29,48]. This regimen has generally established the gold standard by default, given that there is no randomized data to support the use of any other regimen, with the exception of the experience with ECHELON-2 in systemic anaplastic large T-cell lymphoma.

In patients with CD30-positive ALK (+), the ECHELON-2 trial showed that Brentuximab vedotin plus cyclophosphamide, doxorubicin, and prednisone (A + CHP) was associated with significantly higher 5-year PFS compared to patients with CHOP (51.4% vs. 43%), and 5-year OS (70.1% vs. 61%), respectively. These findings suggest that frontline treatment of patients with PTCL with A + CHP continues to provide a clinically meaningful improvement in PFS and OS versus CHOP, with a manageable safety profile [49]. They also showed an improved PFS with A + CHP treatment compared to CHOP in patients with ALK(+) (HR = 0.40, 95% CI: 0.17 to 0.98) and ALK(-) (HR = 0.58, 95% CI: 0.40 to 86). In terms of OS, A + CHP was comparable to CHOP in patients with ALK (+) (HR = 0.48, 95% CI: 0.15 to 1.40) and ALK (−) (HR = 0.71, 95% CI: 0.44 to 1.12) [49].

Regardless, relapse rates are substantial, and long-term outcomes remain low [5]. Furthermore, the overall response rates are generally poor, being in the 50–60% range across the subtypes studied.

The available data suggest that more intensive chemotherapy regimens do not enhance outcomes in patients with PTCL-NOS [50]. In Algeria, patients with advanced-stage PTCL-NOS were treated with CHOP-21, CHOEP-21, COP, and Hyper-CVAD alternating with high-dose methotrexate, and cytarabine was frontline therapy. Similar to other trials, complete response (CR), partial response (PR), and stable disease (SD) were reported in the majority of these cases, although relapse rates were still significant with PFS approximately historical experiences for CHOP-based chemotherapy. Only a few cases reported progressed disease (PD) or mortality [27]. The majority of participants in this study were male (56.7%), with a mean age at diagnosis of 55.1 years (range 21.2 to 82.1). Sixty percent of patients had at least one comorbidity when they were diagnosed. At the time of diagnosis, the majority of patients had advanced disease (81.3% in stages 3 or 4). The most common PTCL types were nodal (45.8%) and leukemic (20.0%), with the most common subtypes being adult T-cell leukemia/lymphoma stage 4 (16.9%) and peripheral TCL—not otherwise specified (NOS) stage 4 (13.6%). The most widely utilized first-line therapies were CHOP-21 (18/44; 40.9%), CHOEP-21 (11/44; 25.0%), and COP (5/44; 11.4%). Despite spurious results seen in one small study after another suggesting improvement in one trial parameter over another, these smaller non-randomized studies are repleate with many confounding factors, and warrant further well-designed randomized studies are needed to confirm superiority of one regimen over another.

In a retrospective, unpublished study, we analyzed the clinical features and outcomes of 99 patients with peripheral T-cell lymphoma treated at two centers in Saudi Arabia. The median patient age was 51 years, with the most common subtype being PTCL-NOS at 38%, followed by ALCL at 34%, NKTCL at 15%, and AITC at 7%. The majority of patients presented with advanced-stage disease (>75%) and had a high International Prognostic Index (IPI) score. The most common frontline therapies were CHOP (36%), CHEOP (16%), and Brentuximab-CHP (7%), resulting in an overall response rate of 54%. While 14% of patients underwent consolidative stem cell transplant, the median progression-free and overall survival were 13 and 24 months, respectively, with 5-year PFS and OS rates of 30% and 44%.

Chemotherapy regimens such as DeVIC, DICE, GDP, VIPD, LVP, GELOX, SMILE, and AspaMetDex have been used to treat ENKTCL-NT, given the penchant for acquired drug resistance seen with CHOP. DeVIC and GELOX are recommended for the early stages of nasal disease [51,52]. SMILE is used for advanced/refractory cases, achieving suboptimal results, but comes with high toxicity risks such as myelosuppression, infections, and mortality [53]. Platinum-containing chemotherapy regimens cause neutropenia and mucositis in patients subjected to RT (50Gy) [54].

## 4. Adult T-Cell Leukemia/Lymphoma (ATLL)

### 4.1. Epidemiology and Risk Factors

ATLL is a heterogeneous, intractable tumor of mature T cells. Human T-cell leukemia virus type I (HTLV-1), which is endemic in several regions, including Romania, central Australia, the Far East, the ME, central Africa, Central and South America, and the southwest region of Japan, is the causative agent [55,56]. The global incidence of ATL is increasing, even in nonendemic areas, due to the migration of people across countries. Patients with ATLL account for 9.6% of all T-cell lymphomas [10]. The high prevalence of ATLL in regions with a high prevalence of HTLV-1 carriers led to the discovery of the relationship between ATLL and HTLV-1 virus since all patients had antibodies against the virus. HTLV-1 was the first retrovirus to be directly linked to human malignancy when it was shown that it immortalizes human T-cells in vitro, which led to the identification of monoclonal integration of HTLV-1 proviral DNA in leukemia cells [57,58].

The highest prevalence of ATLL was found in Asia (25%), followed by North America (2%) and Europe (1%). The male-to-female prevalence of ATLL was 1.2–1.35:1, with a mean age at diagnosis of 57.1 years (range, 24–92 years) [59,60].

Lactation, gender, needle sharing, and blood transfusion are among the ways that the virus may spread. In Kuwait, Egypt, Saudi Arabia, and Lebanon, the reported prevalence of HTLV-1 infection among healthy individuals is very low, at less than 0.1% [61]. Some regions, however, have a much higher HTLV-1 infection rate than others. According to various studies, the prevalence of HTLV-1 infection in the northeast province of Iran ranges from 2 to 3% in the community-based population, 1.7–12% in cross-sectional studies, and 0.34 to 0.77% in blood donors [62,63,64,65,66,67,68].

In the few studies performed in other ME countries (Table 2), the prevalence of HTLV-I infection among healthy subjects varied from 0% to 0.06% in Lebanon, 0.009–0.06% in Saudi Arabia, 0.02% in Egypt and Kuwait [69,70], 1.7% in Jordan [35], and 0.6% in Oman [71]. Within the 276,000 blood donations tested in Israel, just 0.0018% were found to be HTLV-1 seropositive. However, a relatively high prevalence of infection (over 20%) has been observed among an isolated population of Jews originally from the Iranian city of Mashhad [72].

While several clinical studies have been conducted on ATLL patients in the ME [73,74], no epidemiological studies on the incidence or prevalence of ATLL were found in the regional literature. There are several case reports of ATLL, most of which were from Mashhad [75,76].

**Table 2:** Prevalence of HTLV-1 in the ME.

Study ID	Country	Province or City	Population	Study Year	Sample Size	Age	Male-to-Female Ratio	Lab Technique	Prevalence
Zaki et al., 2007 [77]	Egypt	Cairo	Blood donors	2005–2006	90	29.3 ± 6.5	6.50	ELISA, PCR	1.11
Ibrahim et al., 2020 [78]	Iraq	Seven provinces	Blood donors	2015	15,239	36 (20–57)	NA	ELISA, CMIA	0.26
Souan et al., 2016 [79]	Jordan	Amman	Blood donors	2009–2013	62,933	18–60	NA	ELISA	-
Ameen et al., 2005 [80]	Kuwait	Nationwide	Blood donors	2002	12,798	30	NA	ELISA, CMIA	0.01
Tamim et al., 2004 [81]	Lebanon	Nationwide	Blood donors	2001–2002	3529	30.0 ± 8.9	22.80	ELISA, WB, PCR	0.06 (WB), 0.03 (PCR)
Fawzi et al., 2006 [82]	Qatar	Nationwide	Blood donors	1991–2003	124,266	NA	NA	ELISA, WB	0.0002
Aabdien et al., 2020 [83]	Qatar	Doha	Blood donors	2013–2017	190,509	NA	10.03	CMIA, LIA	0.18
Taha et al., 2003 [84]	Saudi Arabia	Al-Khobar	Blood donors	1995–2001	23,493	33.8	NA	ELISA, WB	0.05
Fawaz et al., 2005 [85]	Saudi Arabia	Dammam	Blood donors	1998–2001	13,443	NA	NA	ELISA, WB	0.06
Balkhy et al., 2004 [86]	Saudi Arabia	Riyadh	Blood donors	1999–2001	24,654	NA	NA	ELISA, WB	0.004
Ul-Hassan et al., 2004 [87]	Saudi Arabia	Al-Hasa	Blood donors	1997–2003	47,426	18–55	NA	ELISA, WB	0.01
AlMutairi et al., 2016 [88]	Saudi Arabia	Al-Baha	Blood donors	2009–2011	2807	16–66	NA	ELISA	0.04
Almaiman et al., 2018 [89]	Saudi Arabia	Qassim/Unaizah	Blood donors	2013–2016	9460	31.4 ± 8.7	26.82	ELISA, PCR	0.10
Sarah et al., 2016 [90]	Saudi Arabia	Hail	Blood donors	2014–2016	361	<20 to >50	NA	ELISA	2.22
Alaidarous et al., 2018 [91]	Saudi Arabia	Majmaah	Blood donors	2015–2017	3028	18–61	44.6	ELISA	0.20
Pourkarim et al., 2005 [92]	Iran	Busher (Southwest)	Hemodialysis patients	2003	101	NA	NA	ELISA, WB	0
Karimi et al., 2006 [93]	Iran	Charmahal and Bakhtiari (Southwest)	Hemodialysis patients	2005	107	65 (18–90)	NA	ELISA, WB	6.54
Abedi et al., 2009 [94]	Iran	Hormozgan (South)	Hemodialysis patients	2007–2008	40	NA	NA	ELISA, WB	0
Ardalan et al., 2013 [95]	Iran	Kurdistan (Sanandaj, West)	Hemodialysis patients	2010	65	45.1	NA	ELISA, WB	0
Ghaffari et al., 2013 [96]	Iran	Mazandaran (Sari and Ghaemshahr, North)	Hemodialysis patients	2011	160	59.1 ± 14.7	1.0	ELISA, WB	0.63
Ahmadi et al., 2015 [97]	Iran	Razavi Khorasan (Mashhad, Northeast)	Hemodialysis patients	2009–2010	135	males: (43.5 ± 12.5), females: (50.5 ± 13.2)	0.99	ELISA, PCR	5.93
Hedayati-Moghaddam et al., 2019 [98]	Iran	Razavi Khorasan (Neyshabour, Northeast)	Hemodialysis patients	2012	138	53.3 ± 17.9	1.23	ELISA, WB	14.49
Ziaee et al., 2014 [99]	Iran	South Khorasan (Birjand, East)	Hemodialysis patients	2010	41	54.9 ± 16.5	2.15	ELISA, WB	2.44
Yazdani et al., 2018 [100]	Iran	Tehran (North)	Hemodialysis patients	2018	150	63.6 ± 13.4	1.34	ELISA, WB	0.67
Pourkarim et al., 2005 [92]	Iran	Busher (Southwest)	Thalassemia patients	2003	455	NA	NA	ELISA, WB	3.08
Karimi et al., 2006 [93]	Iran	Charmahal and Bakhtiari (Southwest)	Thalassemia patients	2005	250	25 (1–45)	NA	ELISA, WB	6.8
Arjmandi et al., 2003 [101]	Iran	Fars (Shiraz, South)	Thalassemia patients	2003	200	NA	NA	ELISA	3
Moradi et al., 2008 [102]	Iran	Golestan (Gorgan, North)	Thalassemia patients	2004–2005	181	14.1 ± 6.5	1.06	ELISA, WB	4.42
Pourkarim et al., 2005 [92]	Iran	Busher (Southwest)	Hemophilia patients	2003	86	NA	NA	ELISA, WB	0
ZIAEI et al., 2007 [103]	Iran	South Khorasan (East)	Hemophilia patients	NA	80	21.3 ± 12.1	25.67	ELISA, WB	1.25
Rostamzadeh et al., 2016 [104]	Iran	West Azerbaijan (Northwest)	Hemophilia patients	NA	50	10.3	6.14	ELISA, WB	0

### 4.2. Available Treatments

ATLL treatment approaches are classified by the patient’s clinical presentation. Chemotherapy is often used for patients with aggressive forms such as acute, lymphoma, or unfavorable chronic types. Zidovudine (AZT), a pyrimidine synthetic analog belonging to the class of nucleoside reverse transcriptase inhibitors (NRTIs), is recommended for first-line therapy for all clinical subtypes of ATLL in combination with interferon-alpha (IFN-α), with concomitant chemotherapy reserved for the lymphoma subtype. The combination of AZT and IFN-α is the gold standard for treating leukemia, and survival with antiviral treatment alone is shorter [105,106].

Patients who have smoldering and favorable chronic types are not often subjected to specialized therapies. When applied to these types of lesions, NB-UVB phototherapy is effective for surface lesions, while PUVA is effective for infiltrating lesions [107,108]. According to a recent study, patients with cutaneous involvement and the smoldering type of ATLL responded better to treatments with phototherapy plus etoposide [109]. Like chronic lymphocytic leukemia (CLL) and smoldering myeloma, the favorable chronic and smoldering variants of ATL are thought to be less aggressive and, as such, should be monitored until possible disease progression. Similar to the unfavorable chronic type, chemotherapy treatment for the smoldering form worsens the prognosis [110].

Since its introduction in 1995, AZT and IFN antiviral therapy have been widely utilized as an alternate treatment for ATLL [111]. All patients with chronic and smoldering forms who were initially treated with AZT/IFN survived for more than five years, as shown in a meta-analysis. The 5-year survival was 82% in acute patients treated initially with antivirals, which had a complete response [106]. The combination of AZT and IFN-α was proposed as the gold standard and first-line treatment for leukemic types of ATLL. Chemotherapy should be administered only if antivirals fail to ameliorate the disease [112].

A clinical study conducted in Iran found that AZT/IFN therapy resulted in a high response rate and increased survival with few adverse effects. Further, the authors found that the VEGF plasma levels and HTLV-I proviral load were both greater in ATLL patients than in asymptomatic carriers. In addition, they demonstrated that AZT/IFN treatment decreased HTLV-I proviral load and, more critically, VEGF plasma levels, indicating a possible antiangiogenic impact of this therapy [73]. Another Iranian study by the same group reported a 100% response rate among newly diagnosed ATLL patients treated with arsenic/interferon therapy. Arsenic/IFN/zidovudine therapy sharply diminished IL-10 transcript and serum levels concomitant with a decrease in IL-4 and increases in IFN-γ and IL-2 mRNA [73]. They concluded that the treatment was safe and effective, with moderate toxicity and no relapses [74].

Several chemotherapy regimens have been tested in clinical trials organized by the “Japanese Clinical Oncology Group (JCOG)”。 However, the median survival time varied from 6.5 to 8.5 months [113]. When compared to the biweekly CHOP regimen, the VCAP-AMP-VECP regimen (vincristine, cyclophosphamide, doxorubicin, prednisone–doxorubicin, ranimustine, prednisone–vindesine, etoposide, carboplatin, prednisone) had better results for aggressive clinical forms. Nonetheless, CHOP regimens are favored because of the severe toxicity of the other regimen, particularly in individuals over the age of 70 [114].

Hyper-CVAD is suitable as an alternate regimen, given that some of these regimens are unavailable in the US [115]. As CNS involvement is common in aggressive types (10–25%), intrathecal prophylaxis is suggested [116]. Prophylaxis for strongyloidiasis and pneumocystis pneumonia, among others, and TB screening are also indicated for patients with the severe forms since they are immunocompromised and at high risk for fatal opportunistic infections. Calcium levels should be monitored and controlled for the possibility of tumor lysis syndrome [117].

To enhance these patients’ prognosis, autologous and allogeneic bone marrow transplantation (BMT) has been performed in ATLL. Infections and relapses are common after autologous BMT, making it appear like it is not worth the risk [118]. Several studies have linked allogeneic BMT to increased survival, particularly when myeloablative regimens are used. Its high mortality rate, however, has prevented widespread usage [119].

According to the literature, about 15% of patients treated with a decreased-intensity conditioning regimen have a potentially curative outcome. This is likely attributable to the graft-against-tumor effect [119]. In a retrospective investigation, the 3-year survival rate for 386 Japanese patients who had allogeneic BMT with any induction method was 33%. Having an unrelated donor, a disease without complete remission at the time of BMT, male gender, and age > 50 years were all linked with a poor prognosis [120].

### 4.3. Biological Treatments

Mogamulizumab (KW-0761, Poteligeo): Skin-homing memory T (Th17) cells, interleukin-17 (IL-17) producing T (Th17) cells, regulatory T (Treg) cells, and T-helper type 2 (Th2) cells all express CCR4, a chemokine receptor with a crucial function in immune cell trafficking. Ninety percent of ATLL patients have leukemic cells that express CCR4 on their surface [121]. Anti-CCR4 monoclonal antibody mogamulizumab has been investigated for use in individuals with ATLL who also suffer from CCR4 disease. The overall response rate (ORR) was 50% (95% CI, 0.30–0.70), and the median OS and PFS were 13.7 and 5.2 months, respectively [121]. Both infusion reactions (89%) and skin rashes (63%), the most prevalent side effects, were reversible and manageable. It has been shown that the use of mogamulizumab before ASCT for the treatment of ATLL increases the risk of developing severe acute graft-versus-host disease (GVHD) by 64% (95% CI, 40.7–80.1%) for grades II-IV, with a cumulative incidence of transplantation-related mortality of 49% (95% CI, 27–67.8%) [122].

PJ-34: Clinical studies of PJ-34, a poly (ADP-ribose) polymerase (PARP) inhibitor, for the treatment of solid tumors, have shown promising results, suggesting that PJ-34 therapy may be beneficial for certain ATLL patients; however, HTLV-1-transformed MT2 cells are resistant to treatment [123]. Efforts are being made to investigate the potential use of this molecule in the future treatment of ATLL.

## 5. Cutaneous T-Cell Lymphoma

### 5.1. Mycosis Fungoides and Sezary Syndrome (SS)

#### Mycosis Fungoides/SS

The incidence of MF is the highest among the cutaneous lymphomas, ranging from 9 to 12 cases per 1,000,000 people per year [124,125]. The prognosis is usually related to the stage of the disease, as is the case with most forms of malignant disease. The majority of cases manifest as persistent asymptomatic patches in sun-protected areas. It is common for patients to neglect the eruption until it gets more severe and itchy. Diagnosis typically occurs between 1 and 8 years (median = 3) after the eruption initially appears [126]. Despite this, the prognosis is quite good for over 75% of patients since they are diagnosed at an early stage [127].

MF is rare in the ME; thus, there is a limited number of studies discussing its burden in the region. The majority of the published studies are arising from Saudi Arabia, where MF is regarded as the third most common skin cancer [128,129,130,131,132,133]. Other scattered studies from UAE [19,134], Turkey [135], Kuwait [136], Iran [137], and Egypt [138] have also been published (Table 3). The mean age at diagnosis was found to be comparable to the international figures. Moreover, male predominance was reported in the majority of the studies. Only a few studies reported similar or higher prevalence in females. The time from disease onset to diagnosis was very long in the ME, ranging from 16 to 30 years. A Kuwaiti study showed that the prevalence of MF was significantly higher in the Arabs compared to non-Arabs (0.66 vs. 0.15 per 100,000 per year; risk ratio [RR] = 4.4; 95% CI: 2.9–6.6) [136]. The prevalence of Sezary syndrome ranged between 3 and 5%, and the majority of the patients presented at the early stages of the disease [134,135,138,139].

In terms of treatment options and outcomes, AlGhamdi et al. found that 51.2% of MF Saudi patients received topical corticosteroids, 48.8% received narrow-band UVB, 20.9% received NBUVB and acitretin, 14% had oral psoralen with UVA, 4.7% received systemic chemotherapy, and 2.3% received PUVA and acitretin. They also reported that 48.6% of patients had partial remission, 26.6% did not respond, and 22.9% had complete remission [140]. Similarly, another Saudi study demonstrated that 31.5% of the patients showed improvement, 10.5% showed deterioration, and 10.5% had no change in their disease status. The rest of the patients were lost to follow-up [141].

In Egypt, it was reported that phototherapy was effective in 86.7% of patients with MF, with a success rate of 66.7% for NBUVB and 80% for PUVA [142]. Another Egyptian study showed that the ORR was significantly higher in the PUVA group compared to doxycycline (50% vs. 11%; *p* = 0.016). This response was associated with a significant reduction in CD3 expression, histopathology score, CAILS, and mSWAT; however, this reduction also favored the PUVA over the doxycycline [143]. Likewise, the most commonly used treatment modality in Turkey was PUVA (91%), resulting in 80.4% CR in early-stage patients. Combined with INF-α, it resulted in 33.3% CR in late and 57% in early stages. For late stages, the preferred treatment was systemic therapies such as fludarabine, gemcitabine, and pentostatin [135].

**Table 3:** Burden of MF/SS in the ME.

Study ID	Country	Sample Size	Duration	Prevalence	Age	Male:Female	CD30 Positive	Patchy Infiltration
Mufti S. 2013 [141]	Saudi Arabia	151	1995–2012	12.5%	44.6	3:1	57.89%	89.4%
Al-Maghrabi et al., 2004 [130]	Saudi Arabia	193	1990–2003	1%	70s	Males	NA	NA
Arafah et al., 2012 [144]	Saudi Arabia	58	2002–2006	-	5–74	1.9:1	NA	65.51%
AL-Ghamdi HS 2020 [128]	Saudi Arabia	124	2010–2019	10.5%	58	2.25:1	NA	NA
Albasri and Borhan 2018 [131]	Saudi Arabia	202	2006–2017	6.8%	30.7	3.7:1	NA	NA
Al-Dawsari et al., 2016 [132]	Saudi Arabia	204	1995–2014	11%	52 (22–82)	2.29:1	NA	NA
Alwunais and Ahmad 2015 [133]	Saudi Arabia	27	2008–2014	14.8%	53	1:1	NA	NA
Alojail et al., 2020 [139]	Saudi Arabia	34	2011–2016	-	>30 (50%)	3.12:1	100%	NA
Binamer Y. 2017 [144]	Saudi Arabia	125	2010–2016	-	41 (27–54)	1.35:1	0.8%	22.4%
AlGhamdi et al., 2012 [140]	Saudi Arabia	43	2000–2006		33.5 (5–74)	2:1	NA	48.8%
Mowafak et al., 2021 [134]	UAE	40	2013–2019	-	47	0.8:1	NA	67.5%
Castella et al., 2001 [19]	UAE	208	1988–1999	0.96%	62	1:1	NA	NA
Al-Tarawneh et al., 2018 [145]	Jordan	63	2000–2015	-	45 (11–80)	2.15:1	NA	39.6%
Haddadin W. 2005 [146]	Jordan	347	2001–2003	3.67%	48.5 ± 21.7	2.33:1	NA	NA
Aladily et al., 2020 [35]	Jordan	4189	2014–2019	4.1%	42	1.07:1	NA	NA
Alsaleh et al., 2010 [136]	Kuwait	193	1991–2006	0.43 cases per 100,000	35.20 ± 14.37	2:1	NA	67%
Anadolu et al., 2005 [135]	Turkey	113	1984–2001	-	45.6 ± 15.8	1.05:1	NA	46.9%
Naeini et al., 2012 [137]	Iran	25	-	-	41.06	0.6:1	NA	38.9%
Abdel-Halim et al., 2015 [138]	Egypt	100	2012–2013	16%	21.31 ± 10.88	0.3:1	NA	NA
Hassab-El-Naby et al., 2013 [142]	Egypt	27	2004–2011	-	35.39 ± 13.13	2:1	NA	NA
Nadia Boudjera et al., 2022 [27]	Algeria	60	2017–2019	1.7%	55.1 (21.2–82.1)	1.3:1	NA	NA
Humam et al., 2016 [40]	Yemen	170	2008–2013	1.76%	65	Females	NA	NA

## 6. Future Perspectives and Unmet Needs

Given this well-established variation in geographic distribution and sometimes a natural history of the TPCL, it is clear we need to broaden and expand regional registries to better understand this complex disease. PTCL is not a single disease, as underscored above, but it represents a collection of over 36 discrete entities, each with its own nuances and responsiveness to various treatments. Appreciating these differences is essential to prioritizing resources to manage the disease in a locoregional manner.

As such, it is one of the objectives of creating a national and regional unified registry for PTCLs to help us better understand the epidemiology since the regional literature may not accurately reflect the PTCLs in the ME. Further, we are working to create outreach campaigns to rural hospitals to raise awareness and understanding of recent advances in PTCLs to mitigate difficulties in diagnosing and treating the elderly and unfit PTCL population. We acknowledge many unmet needs in the management of PTCLs, both globally and regionally.

Presently, the data related to the burden of PTCLs and regional epidemiology are scarce. The prompt transfer of patients with PTCLs to a tertiary hospital for specialized treatment necessitates the implementation of an electronic referral system. Patients ineligible for allo-HCT continue to be treated at community hospitals that may lack the necessary resources to provide adequate care for them. Due to a lack of molecular testing capabilities, many hospitals that treat patients with PTCLs have to transfer samples to a reference laboratory abroad, which lengthens the time it takes to obtain findings. Patients’ access to novel agents has been limited by the high price of newer PTCL therapeutic agents and the absence of PTCL clinical trials in the area. Consequently, we suggest a more extensive collaboration between pharmaceutical industries and government health sectors to expand the number of clinical studies.

Increasing the number of clinical trials, especially those funded by the pharmaceutical industry, grants, and the government, would benefit medical practice and provide patients with earlier access to effective and novel drugs. In addition, clinical trials will shed light on the appropriate agents for the ME population. Governmental efforts to include the novel agents under the umbrella of health insurance will facilitate delivering these agents to patients in need.

Although certain facilities in the ME have cutting-edge labs for diagnostic and molecular genetic testing, this is not the case for the vast majority of facilities. This shortage may compromise the delivery of timely and appropriate care. We would thus propose the creation of central reference labs at both the regional and national levels, supported by highly trained experts in conducting molecular testing and profiling, which may result in reducing the resources spent on external tests and ensuring that the findings are consistent. To further facilitate risk stratification and treatment decision-making, we propose an algorithmic diagnostic method for the diagnosis and categorization of TCLs based on the WHO classification, as well as a workup algorithm to guide clinicians.

## 7. Conclusions

PTCLs are a rare and heterogeneous group of diseases with much still unknown. Despite initial improvements, a large proportion of patients eventually relapse. Some PTCLs have well-understood etiologies, such as ATLL and HTLV-1; however, despite this knowledge, treatment of the original trigger of carcinogenesis does not enhance prognosis. High rates of cell division and mutation lead to R/R that is resistant to treatment, even when treatments are targeted at specific gene mutations and manifestations of malignancy subtypes.

We need to learn more about the biochemical, genetic, histological, and clinical elements of the disease’s course in order to provide patients with more effective therapy options and, eventually, improve their chances of survival. Clinical trials are frequently regarded as the first line of treatment for TCLs because current chemotherapy regimens are not as effective or well tolerated by patients as we would desire. This highlights the importance of basic and clinical research in assisting us in combating these forms of lymphomas while avoiding the unfavorable side effects of toxic regimens for our patients. Table 4 summarizes the difference in the frequency, survival, and treatment differences between indolent T-cell lymphoma (ITCK) and aggressive T-cell lymphomas.

The prevalence of PTCLs in the ME is not extensively documented, as most studies have been undertaken in Western nations. However, current research indicates that the prevalence of TCLs in the ME is comparable to that of Western countries. Furthermore, some research suggests that the prevalence of T-cell lymphomas in ME may be higher than it was a decade ago. There is a very limited number of clinical trials arising from the ME regarding the management of PTCLs; moreover, there is a lack of real-world evidence and management consensus in the region. The absence of PTCL registries in the region is one of the leading causes of the limited evidence arising from the region.

The diagnosis of PTCLs in the ME is sometimes complex and necessitates specialized investigations, including biopsies, imaging, and blood tests. Because a biopsy can detect the presence of abnormal T-cells, it is the most often used diagnostic method for TCLs in the ME. Furthermore, imaging tests such as PET/CT, MRI, and CT are commonly used to determine the location and size of lymphoma tumors. In this region, TCL profiling and molecular testing are not frequently employed.

The treatment of PTCLs in the ME is often complex and may include chemotherapy, radiation therapy, and immunotherapy. Chemotherapy is the most common treatment option for TCLs in the ME and is usually used in combination with other treatments, such as radiation therapy or immunotherapy. Radiation therapy can be used to target areas of the body affected by PTCLs and reduce the size of the tumor. However, it is clear that the development of novel/novel drug combinations can produce benefits that appear as effective as traditional chemotherapy. Regrettably, the ME has not been at the forefront of clinical trials in these and related types of lymphoma. Our active engagement in cutting-edge research will improve access to state-of-the-art drugs, which many lines of evidence suggest may be associated with superior outcomes. In addition, enrollment of patients in clinical trials affords the region an opportunity to capture information in a comprehensive fashion, further redounding our ability to define and optimize the regional care of patients with PTCL. These research efforts need to include not only access to new small-targeted drugs but also to the latest advances in immunotherapy, including adoptive cellular therapies. By drawing attention to the nuanced differences of PTCL in the Middle East, we look forward to bolstering our research goals in improving access and outcomes of all patients in the ME and beyond.

## Figures and Tables

**Table 1:** Epidemiology of ALCL in the ME.

Study ID	Country	Lymphoma Sample Size	Duration	Type of ALK	Prevalence N (%)	Age	Male:Female
Sherief et al., 2015 [39]	Egypt	142	2004–2012	Both	3 (2.1%)	6.1 ± 2.8	1.7:1
Jalili et al., 2022 [26]	Iran	659	2018–2021	ALK−	3 (0.5%; 5.1% of TCLs)	37.3 ± 11.8	-
Akhtar et al., 2009 [17]	Saudi Arabia	385	1988–2007	Both	8 (2.08%; 17.4% if TCLs)	55	1.3:1
Nadia Boudjera et al., 2022 [27]	Algeria	60	2017–2019	Both	10 (16.67%)	55.1 (21.2–82.1)	1.3:1
Castella et al., 2001 [19]	UAE	208	1988–1999	Both	4 (1.92%)	59 ± 49	2:1
Mjali et al., 2021 [36]	Iraq	385	2012–2020	Both	5 (1.3%)	47	0.67:1
Humam et al., 2016 [40]	Yemen	170	2008–2013	Both	7 (4.12%; 33.3% of TCLs)	60	1.33:1
Aladily et al., 2020 [35]	Jordan	4189	2014–2019	Both	71 (1.7%: 8% TCLs)	39	1.63:1
SAĞLAM et al., 2018 [28]	Turkey	4239	2000–2017	Both	106 (2.5%; 14.5% of TCLs)	39 (25–55)	NA

**Table 4:** Frequency, survival, and treatment differences between indolent T-cell lymphoma (ITCK) and aggressive T-cell lymphomas.

#	Prognosis	OS	Percentage	Treatment
1	ITCL	5 years	76%	Active monitoring/less aggressive therapy
2	PTCL-NOS	5 years	32%	CHOP or CHOEP chemotherapy Allogeneic stem cell transplantation Zidovudine plus interferon-α
3	AITL	3 years	67.5%	
4	ALCL ALK+	3 years	89.8%	
5	ALCL ALK−	3 years	62.1%	
6	Acute ATL	6 months		
Refs.: [32,147,148]				

## References

[1] Varghese M.T., Alsubait S. (2022). T-Cell lymphoma. StatPearls [Internet].

[2] Alaggio R., Amador C., Anagnostopoulos I., Attygalle A.D., Araujo I.B.D.O., Berti E., Bhagat G., Borges A.M., Boyer D., Calaminici M. (2022). The 5th edition of the World Health Organization classification of haematolymphoid tumours: Lymphoid neoplasms. Leukemia.

[3] Park H.S., McIntosh L., Braschi-Amirfarzan M., Shinagare A.B., Krajewski K.M. (2017). T-Cell Non-Hodgkin Lymphomas: Spectrum of Disease and the Role of Imaging in the Management of Common Subtypes. Korean J. Radiol..

[4] Campo E., Jaffe E.S., Cook J.R., Quintanilla-Martinez L., Swerdlow S.H., Anderson K.C., Brousset P., Cerroni L., de Leval L., Dirnhofer S. (2022). The international consensus classification of mature lymphoid neoplasms: A report from the clinical advisory committee. Blood J. Am. Soc. Hematol..

[5] Broccoli A., Zinzani P.L. (2017). Peripheral T-cell lymphoma, not otherwise specified. Blood J. Am. Soc. Hematol..

[6] Carson K.R., Horwitz S.M., Pinter-Brown L.C., Rosen S.T., Pro B., Hsi E.D., Federico M., Gisselbrecht C., Schwartz M., Bellm L.A. (2017). A prospective cohort study of patients with peripheral T-cell lymphoma in the United States. Cancer.

[7] Foss F.M., Zinzani P.L., Vose J.M., Gascoyne R.D., Rosen S.T., Tobinai K. (2011). Peripheral T-cell lymphoma. Blood J. Am. Soc. Hematol..

[8] Bellei M., Nabhan C., Pesce E.A., Conte L., Vose J.M., Foss F., Federico M. (2015). The Value and Relevance of the T Cell Lymphoma Registries and International Collaborations: The Case of COMPLETE and the T-Cell Project. Curr. Hematol. Malig. Rep..

[9] Hildyard C.A.T., Shiekh S., Browning J.A.B., Collins G.P. (2017). Toward a Biology-Driven Treatment Strategy for Peripheral T-cell Lymphoma. Clin. Med. Insights Blood Disord..

[10] Vose J., Armitage J., Weisenburger D., International T-Cell Lymphoma Project (2008). International peripheral T-cell and natural killer/T-cell lymphoma study: Pathology findings and clinical outcomes. J. Clin. Oncol. Off. J. Am. Soc. Clin. Oncol..

[11] Adams S.V., Newcomb P.A., Shustov A.R. (2016). Racial Patterns of Peripheral T-Cell Lymphoma Incidence and Survival in the United States. J. Clin. Oncol. Off. J. Am. Soc. Clin. Oncol..

[12] Phan A., Veldman R., Lechowicz M.J. (2016). T-cell Lymphoma Epidemiology: The Known and Unknown. Curr. Hematol. Malig. Rep..

[13] Weisenburger D.D., Savage K.J., Harris N.L., Gascoyne R.D., Jaffe E.S., MacLennan K.A., Rüdiger T., Pileri S., Nakamura S., Nathwani B. (2011). Peripheral T-cell lymphoma, not otherwise specified: A report of 340 cases from the International Peripheral T-cell Lymphoma Project. Blood.

[14] Wang S.S., Flowers C.R., Kadin M.E., Chang E.T., Hughes A.M., Ansell S.M., Feldman A.L., Lightfoot T., Boffetta P., Melbye M. (2014). Medical history, lifestyle, family history, and occupational risk factors for peripheral T-cell lymphomas: The InterLymph Non-Hodgkin Lymphoma Subtypes Project. J. Natl. Cancer Inst. Monogr..

[15] Perry A.M., Diebold J., Nathwani B.N., MacLennan K.A., Müller-Hermelink H.K., Bast M., Boilesen E., Armitage J.O., Weisenburger D.D. (2016). Relative frequency of non-Hodgkin lymphoma subtypes in selected centres in North Africa, the middle east and India: A review of 971 cases. Br. J. Haematol..

[16] Otrock Z.K., Saab J., Aftimos G., Nasr F., Farhat F.S., Khairallah S., Abadjian G., Ghosn M., Sidani H., Ibrahim A. (2013). A collaborative nationwide lymphoma study in Lebanon: Incidence of various subtypes and analysis of associations with viruses. Pathol. Oncol. Res..

[17] Akhtar S.S., Haque I.U., Wafa S.M., El-Saka H., Saroor A.M., Nadrah H.M. (2009). Malignant lymphoma in Al-Qassim, Saudi Arabia, reclassified according to the WHO classification. Saudi Med. J..

[18] Almasri N.M., Habashneh M.A., Khalidi H.S. (2004). Non-Hodgkin lymphoma in Jordan. Types and patterns of 111 cases classified according to the WHO classification of hematological malignancies. Saudi Med. J..

[19] Castella A., Joshi S., Raaschou T., Mason N. (2001). Pattern of malignant lymphoma in the United Arab Emirates--a histopathologic and immunologic study in 208 native patients. Acta Oncol..

[20] Isikdogan A., Ayyildiz O., Buyukcelik A., Arslan A., Tiftik N., Buyukbayram H., Muftuoglu E. (2004). Non-Hodgkin’s lymphoma in southeast Turkey: Clinicopathologic features of 490 cases. Ann. Hematol..

[21] Yaqo R.T., Hughson M.D., Sulayvani F.K., Al-Allawi N.A. (2011). Malignant lymphoma in northern Iraq: A retrospective analysis of 270 cases according to the World Health Organization classification. Indian J. Cancer.

[22] Abdel-Fattah M.M., Yassine O.G. (2007). Non-Hodgkin’s lymphomas in Alexandria, Egypt; incidence rates and trend study (1995–2004). Eur. J. Cancer Prev. Off. J. Eur. Cancer Prev. Organ. (ECP).

[23] Goldman L., Ezzat S., Mokhtar N., Abdel-Hamid A., Fowler N., Gouda I., Eissa S.A., Abdel-Hamid M., Loffredo C.A. (2009). Viral and non-viral risk factors for non-Hodgkin’s lymphoma in Egypt: Heterogeneity by histological and immunological subtypes. Cancer Causes Control.

[24] Ameen R., Sajnani K.P., Albassami A., Refaat S. (2010). Frequencies of non-Hodgkin’s lymphoma subtypes in Kuwait: Comparisons between different ethnic groups. Ann. Hematol..

[25] Mozaheb Z., Aledavood A., Farzad F. (2011). Distributions of major sub-types of lymphoid malignancies among adults in Mashhad, Iran. Cancer Epidemiol..

[26] Jalili J., Vahedi A., Danandehmehr A., Aynechi P., Esfahani A., Roosta Y., Nateghian H., Ghafouri Asbagh A., Hajihoseinlou F. (2022). Subtype distribution of lymphomas in northwestern Iran: A retrospective analysis of 659 cases according to World Health Organization classification. BMC Cancer.

[27] Belarbi N.B., Bekadja M.A., Abad M.T., Webb D., Grifi F. (2022). Peripheral T-Cell Lymphomas in Algeria: Results from a Multicenter Registry Study. Int. J. Rare Dis. Disord..

[28] Sağlam A., Esin E., Hayran M., Boyraz B., Üner A. (2018). Distribution of lymphomas in Turkey: Data of 4239 cases from a single institution using the WHO classification. Turk. J. Med. Sci..

[29] Al-Zahrani M., Savage K.J. (2017). Peripheral T-Cell Lymphoma, Not Otherwise Specified: A Review of Current Disease Understanding and Therapeutic Approaches. Hematol. Oncol. Clin. N. Am..

[30] Xu P., Yu D., Wang L., Shen Y., Shen Z., Zhao W. (2015). Analysis of prognostic factors and comparison of prognostic scores in peripheral T cell lymphoma, not otherwise specified: A single-institution study of 105 Chinese patients. Ann. Hematol..

[31] Savage K.J., Ferreri A.J., Zinzani P.L., Pileri S.A. (2011). Peripheral T-cell lymphoma–not otherwise specified. Crit. Rev. Oncol. Hematol..

[32] Schmitz N., de Leval L. (2017). How I manage peripheral T-cell lymphoma, not otherwise specified and angioimmunoblastic T-cell lymphoma: Current practice and a glimpse into the future. Br. J. Haematol..

[33] Gurion R., Bernstine H., Domachevsky L., Michelson C., Raanani P., Vidal L., Shochat T., Gafter-Gvili A. (2018). Utility of PET-CT for Evaluation of Patients With Peripheral T-cell Lymphoma. Clin. Lymphoma Myeloma Leuk..

[34] Aljufairi E.A., George S.M., Alshaikh S.A., Radhi A.A., Mohamed R.M. (2018). Spectrum of lymphoma in Bahrain. A retrospective analysis according to the World Health Organization classification. Saudi Med. J..

[35] Aladily T.N., Khreisat W., Ashukhaibi O., Alkhatib S.M., Annab H., Tarawneh M.S., Salman T.S., Abu Farsakh H., Mahgoub R., Bustami N. (2021). The epidemiology of lymphoma in Jordan: A nationwide population study of 4189 cases according to World Health Organization classification system. Hematol. Oncol. Stem Cell Ther..

[36] Mjali A., Oudah A.H., Al-Shammari H.H., Abbas N.T. (2021). Classification of non-Hodgkin lymphoma in the Middle Euphrates Region of Iraq according to the World Health Organization classification. Iraqi J. Hematol..

[37] Miranda R.N., Aladily T.N., Prince H.M., Kanagal-Shamanna R., de Jong D., Fayad L.E., Amin M.B., Haideri N., Bhagat G., Brooks G.S. (2014). Breast implant-associated anaplastic large-cell lymphoma: Long-term follow-up of 60 patients. J. Clin. Oncol. Off. J. Am. Soc. Clin. Oncol..

[38] Morton L.M., Wang S.S., Devesa S.S., Hartge P., Weisenburger D.D., Linet M.S. (2006). Lymphoma incidence patterns by WHO subtype in the United States, 1992–2001. Blood.

[39] Sherief L.M., Elsafy U.R., Abdelkhalek E.R., Kamal N.M., Youssef D.M., Elbehedy R. (2015). Disease patterns of pediatric non-Hodgkin lymphoma: A study from a developing area in Egypt. Mol. Clin. Oncol..

[40] Humam M.A., Al-Nakhbi N.A., Melkat A.A., Almontaser T.M., Binnabhan A. (2016). S Malignant lymphoma in Hadhramout Sector, Yemen: A retrospective study of 170 cases classified according to the WHO classification. J. Curr. Med. Res. Pract..

[41] Pongpruttipan T., Sukpanichnant S., Assanasen T., Wannakrairot P., Boonsakan P., Kanoksil W., Kayasut K., Mitarnun W., Khuhapinant A., Bunworasate U. (2012). Extranodal NK/T-cell Lymphoma, Nasal Type, Includes Cases of Natural Killer Cell and αβ, γδ, and αβ/γδ T-cell Origin. Am. J. Surg. Pathol..

[42] Su Y.J., Wang P.N., Chang H., Shih L.Y., Lin T.L., Kuo M.C., Chuang W.Y., Wu J.H., Tang T.C., Hung Y.S. (2018). Extranodal NK/T-cell lymphoma, nasal type: Clinical features, outcome, and prognostic factors in 101 cases. Eur. J. Haematol..

[43] Jia J., Song Y., Lin N., Liu W., Ping L., Zheng W., Wang X., Xie Y., Tu M., Zhang C. (2016). Clinical features and survival of extranodal natural killer/T cell lymphoma with and without hemophagocytic syndrome. Ann. Hematol..

[44] Yang Q.P., Zhang W.Y., Yu J.B., Zhao S., Xu H., Wang W.Y., Bi C.F., Zuo Z., Wang X.Q., Huang J. (2011). Subtype distribution of lymphomas in Southwest China: Analysis of 6,382 cases using WHO classification in a single institution. Diagn. Pathol..

[45] Motabi I., Alzahrani M., Dada R., Al-Mansour M., Alhashmi H., Kandil M., Sagheir A., Alhejazi A. (2019). Natural Killer/T-Cell Lymphoma: Saudi Lymphoma Group’s Clinical Practice Guidelines for Diagnosis, Management and Follow-up. Saudi J. Med. Med. Sci..

[46] Takahara M., Kumai T., Kishibe K., Nagato T., Harabuchi Y. (2021). Extranodal NK/T-Cell Lymphoma, Nasal Type: Genetic, Biologic, and Clinical Aspects with a Central Focus on Epstein-Barr Virus Relation. Microorganisms.

[47] Sabattini E., Bacci F., Sagramoso C., Pileri S.A. (2010). WHO classification of tumours of haematopoietic and lymphoid tissues in 2008: An overview. Pathologica.

[48] Horwitz S.M., Zelenetz A.D., Gordon L.I., Wierda W.G., Abramson J.S., Advani R.H., Andreadis C.B., Bartlett N., Byrd J.C., Fayad L.E. (2016). NCCN Guidelines Insights: Non-Hodgkin’s Lymphomas, Version 3.2016. J. Natl. Compr. Cancer Netw..

[49] Horwitz S., O’Connor O.A., Pro B., Trümper L., Iyer S., Advani R., Bartlett N.L., Christensen J.H., Morschhauser F., Domingo-Domenech E. (2022). The ECHELON-2 Trial: 5-year results of a randomized, phase III study of brentuximab vedotin with chemotherapy for CD30-positive peripheral T-cell lymphoma. Ann. Oncol. Off. J. Eur. Soc. Med. Oncol..

[50] Mehta N., Maragulia J.C., Moskowitz A., Hamlin P.A., Lunning M.A., Moskowitz C.H., Zelenetz A., Matasar M.J., Sauter C., Goldberg J. (2013). A retrospective analysis of peripheral T-cell lymphoma treated with the intention to transplant in the first remission. Clin. Lymphoma Myeloma Leuk..

[51] Asano N., Kato S., Nakamura S. (2013). Epstein–Barr virus-associated natural killer/T-cell lymphomas. Best Pract. Res. Clin. Haematol..

[52] Wang L., Wang Z.H., Chen X.Q., Li Y.J., Wang K.F., Xia Y.F., Xia Z.J. (2013). First-line combination of gemcitabine, oxaliplatin, and L-asparaginase (GELOX) followed by involved-field radiation therapy for patients with stage IE/IIE extranodal natural killer/T-cell lymphoma. Cancer.

[53] Makita S., Tobinai K. (2017). Clinical Features and Current Optimal Management of Natural Killer/T-Cell Lymphoma. Hematol. Oncol. Clin. N. Am..

[54] Tse E., Kwong Y.L. (2013). How I treat NK/T-cell lymphomas. Blood.

[55] Jabbour M., Tuncer H., Castillo J., Butera J., Roy T., Pojani J., Al-Malki M., Al-Homsi A.S. (2011). Hematopoietic SCT for adult T-cell leukemia/lymphoma: A review. Bone Marrow Transplant..

[56] Gonçalves D.U., Proietti F.A., Ribas J.G., Araújo M.G., Pinheiro S.R., Guedes A.C., Carneiro-Proietti A.B. (2010). Epidemiology, treatment, and prevention of human T-cell leukemia virus type 1-associated diseases. Clin. Microbiol. Rev..

[57] Takatsuki K. (2005). Discovery of adult T-cell leukemia. Retrovirology.

[58] Ohshima K. (2015). Molecular Pathology of Adult T-Cell Leukemia/Lymphoma. Oncology.

[59] Shimoyama M. (1991). Diagnostic criteria and classification of clinical subtypes of adult T-cell leukaemia-lymphoma. A report from the Lymphoma Study Group (1984–1987). Br. J. Haematol..

[60] Kondo T., Kono H., Miyamoto N., Yoshida R., Toki H., Matsumoto I., Hara M., Inoue H., Inatsuki A., Funatsu T. (1989). Age- and sex-specific cumulative rate and risk of ATLL for HTLV-I carriers. Int. J. Cancer.

[61] Proietti F.A., Carneiro-Proietti A.B., Catalan-Soares B.C., Murphy E.L. (2005). Global epidemiology of HTLV-I infection and associated diseases. Oncogene.

[62] Abbaszadegan M.R., Gholamin M., Tabatabaee A., Farid R., Houshmand M., Abbaszadegan M. (2003). Prevalence of human T-lymphotropic virus type 1 among blood donors from Mashhad, Iran. J. Clin. Microbiol..

[63] Khameneh Z.R., Baradaran M., Sepehrvand N. (2008). Survey of the seroprovalence of HTLV I/II in hemodialysis patients and blood donors in Urmia. Saudi J. Kidney Dis. Transplant..

[64] Meytes D., Schochat B., Lee H., Nadel G., Sidi Y., Cerney M., Swanson P., Shaklai M., Kilim Y., Elgat M. (1990). Serological and molecular survey for HTLV-I infection in a high-risk Middle Eastern group. Lancet.

[65] Hedayati-Moghaddam M.R., Fathimoghadam F., Eftekharzadeh Mashhadi I., Soghandi L., Bidkhori H.R. (2011). Epidemiology of HTLV-1 in Neyshabour, Northeast of Iran. Iran. Red. Crescent Med. J..

[66] Azarpazhooh M.R., Hasanpour K., Ghanbari M., Rezaee S.A., Mashkani B., Hedayati-Moghaddam M.R., Valizadeh N., Farid Hosseini R., Foroghipoor M., Soltanifar A. (2012). Human T-lymphotropic virus type 1 prevalence in northeastern Iran, Sabzevar: An epidemiologic-based study and phylogenetic analysis. AIDS Res. Hum. Retroviruses.

[67] Rafatpanah H., Hedayati-Moghaddam M.R., Fathimoghadam F., Bidkhori H.R., Shamsian S.K., Ahmadi S., Sohgandi L., Azarpazhooh M.R., Rezaee S.A., Farid R. (2011). High prevalence of HTLV-I infection in Mashhad, Northeast Iran: A population-based seroepidemiology survey. J. Clin. Virol. Off. Publ. Pan Am. Soc. Clin. Virol..

[68] Safai B., Huang J.L., Boeri E., Farid R., Raafat J., Schutzer P., Ahkami R., Franchini G. (1996). Prevalence of HTLV type I infection in Iran: A serological and genetic study. AIDS Res. Hum. Retroviruses.

[69] Constantine N.T., Fathi Sheba M., Corwin A.L., Danahy R.S., Callahan J.D., Watts D.M. (1991). A serosurvey for HTLV-I among high-risk populations and normal adults in Egypt. Epidemiol. Infect..

[70] Al-Mufti S., Voevodin A., Ahmed S., Al-Hamdan S., Al-Bisher A.A. (1997). Prevalence of human T-cell lymphotropic virus type I infection among volunteer blood donors in Kuwait. J. Acquir. Immune Defic. Syndr. Hum. Retrovirol..

[71] Knox-Macaulay H., McDonald R., al-Busaidy S., Ali N. (1997). HTLV-I seroprevalence in the Sultanate of Oman. Scand. J. Infect. Dis..

[72] Miller M., Achiron A., Shaklai M., Stark P., Maayan S., Hannig H., Hunsmann G., Bodemer W., Shohat B. (1998). Ethnic cluster of HTLV-I infection in Israel among the Mashhadi Jewish population. J. Med. Virol..

[73] Kchour G., Makhoul N.J., Mahmoudi M., Kooshyar M.M., Shirdel A., Rastin M., Rafatpanah H., Tarhini M., Zalloua P.A., Hermine O. (2007). Zidovudine and interferon-alpha treatment induces a high response rate and reduces HTLV-1 proviral load and VEGF plasma levels in patients with adult T-cell leukemia from North East Iran. Leuk. Lymphoma.

[74] Kchour G., Tarhini M., Kooshyar M.M., El Hajj H., Wattel E., Mahmoudi M., Hatoum H., Rahimi H., Maleki M., Rafatpanah H. (2009). Phase 2 study of the efficacy and safety of the combination of arsenic trioxide, interferon alpha, and zidovudine in newly diagnosed chronic adult T-cell leukemia/lymphoma (ATL). Blood.

[75] Sidi Y., Meytes D., Shohat B., Fenig E., Weisbort Y., Lee H., Pinkhas J., Rosenblatt J.D. (1990). Adult T-cell lymphoma in Israeli patients of Iranian origin. Cancer.

[76] Bitar N., Hajj H.E., Houmani Z., Sabbah A., Otrock Z.K., Mahfouz R., Zaatari G., Bazarbachi A. (2009). Adult T-cell leukemia/lymphoma in the Middle East: First report of two cases from Lebanon. Transfusion.

[77] Zaki S.M., Darwish M.M., Mahmoud M.H. (2007). Sporadic carriers of human T-cell lymphotropic virus type 1 among blood donors in Egypt. Egypt. J. Med. Lab. Sci..

[78] Ibrahim A.I., Al-Musawi Y.A., Abdullah A.I. (2020). Seroprevalence of HTLV-type-1 and type-2 among Blood Donors in Some Iraqi Provinces. Indian J. Forensic Med. Toxicol..

[79] Souan L., Tout F., Siag M., Sughayer M.A. (2016). Seroprevalence rates of transfusion-transmitted infections among blood donors in Jordan. J. Infect. Dev. Ctries..

[80] Ameen R., Sanad N., Al-Shemmari S., Siddique I., Chowdhury R.I., Al-Hamdan S., Al-Bashir A. (2005). Prevalence of viral markers among first-time Arab blood donors in Kuwait. Transfusion.

[81] Tamim H., Musharrafieh U., Ramia S., Almawi W.Y., Al-Jisr T., Ayoub T., Nabulsi-Majzoub M., Kazma H., Baz E.K. (2004). Is seroprevalence of HTLV-I/II among blood donors in Lebanon relevant?. Am. J. Infect. Control.

[82] Fawzi Z.O., Al Malki A., Al Mutawa H. (2006). Prevalence of Human T-Lymphotropic Virus (HTLV) Antibodies Among the Donor Population in the State of Qatar. Qatar Med. J..

[83] Aabdien M., Selim N., Himatt S., Hmissi S., Merenkov Z., AlKubaisi N., Abdel-Rahman M.E., Abdelmola A., Khelfa S., Farag E. (2020). Prevalence and trends of transfusion transmissible infections among blood donors in the State of Qatar, 2013–2017. BMC Infect. Dis..

[84] Taha M.A., Bashawri L.A., Ahmed M.S., Ahmed M.A. (2003). Prevalence of antibodies to human T-lymphotropic viruses types I and II among healthy blood donors. Saudi Med. J..

[85] Fawaz N.A., Tamim H., Almawi W.Y. (2005). Low prevalence of antibodies to human T-lymphotropic virus-I/II among blood donors in eastern Saudi Arabia. Am. J. Infect. Control.

[86] Balkhy H.H., Memish Z.A., Abed E., Qasem L., Amer A.B., Masoud S., Hajeer A.H. (2004). Saudi National Guard donor screening for human T cell lymphotropic virus I/II: Time to use molecular biology techniques. Mil. Med..

[87] Ul-Hassan Z., Al-Bahrani A.T., Panhotra B.R. (2004). Prevalence of human T-lymphotropic virus type I and type II antibody among blood donors in Eastern Saudi Arabia. Saudi Med. J..

[88] AlMutairi H.H., AlAhmari M.M., Al-Zahran B.H., Abbas I.S., Al Ghamdi J.A., Raja A Y.A., Sallam T.A. (2016). Prevalence of serological markers and nucleic acid for blood-borne viral infections in blood donors in Al-Baha, Saudi Arabia. J. Infect. Dev. Ctries..

[89] Almaiman A.A., Almaiman S.H. (2018). Evaluation of Blood Donors and transfusion transmitted infections and their association with ABO and Rh Blood groups in Unaizah, Saudi Arabia: A retrospective study. Int. J. Med. Res. Health Sci..

[90] Sarah Y.A.E.G.A., Ali A.L. (2016). Seropositivity of TTIs among blood donors in Hail, Saudi Arabia, from 2014 to 2015. Asian Pac. J. Trop. Dis..

[91] Alaidarous M., Choudhary R.K., Waly M.I., Mir S., Dukhyil A.B., Banawas S.S., Alshehri B.M. (2018). The prevalence of transfusion-transmitted infections and nucleic acid testing among blood donors in Majmaah, Saudi Arabia. J. Infect. Public Health.

[92] Pourkarim M.R., Khamisipour G., Hajiani G., Tahmasebi R., Ardeshirdavani N. (2005). Seroepidemiological investigation of HTLV I, II infection among Busherian multi-transfused patients in 2003. Sci. J. Iran Blood Transfus Organ.

[93] Karimi A., Nafisi M. (2006). Seroprevalence of human T-cell leukemia virus type-1 (HTLV-1) in high risk patients. J. Res. Health Sci..

[94] Abedi F.A.R.S.H.I.D., Yavarian M.A.J.I.D., Shakibzadeh A., Khalvati B., Asadi A.H. (2009). A pilot Seroepidemiologic study of HTLV in thalassemia, hemophilia, and hemodialysed patients in Hormozgan. HMJ.

[95] Ardalan N., Abdi M., Zarif B.R., Amini A., Memary F., Haydari E., Ahmadi A. (2013). Prevalence of human T-lymphotropicvirus types I&II among high risk groups in Sanandaj in 2010. Sci. J. Kurd. Univ. Med. Sci..

[96] Ghaffari J., Ebrahimi M., Makhlough A., Mohammadjafari H., Nazari Z. (2013). Seroepidemiology of human T-cell lymphotropic virus 1 infection in hemodialysis patients: Should we be concerned about it?. Iran. J. Kidney Dis..

[97] Ghezeldasht S.A., Hassannia T., Rafatpanah H., Hekmat R., Valizadeh N., Mobarhan M.G., Rezaee S.A. (2015). Oncogenic Virus Infections in the General Population and End-stage Renal Disease Patients With Special Emphasis on Kaposi’s Sarcoma Associated Herpes Virus (KSHV) in Northeast of Iran. Jundishapur J. Microbiol..

[98] Hedayati-Moghaddam M.R., Fathimoghadam F., Soghandi L., Darrudi A. (2019). High Prevalence of HTLV-1 Infection Among Hemodialysis Patients in Neyshabur, Northeast of Iran. Int. J. Infect..

[99] Ziaee M., Azizee R., Namaei M.H. (2014). Prevalence of HCV infection in hemodialysis patients of South Khorasan in comparison With HBV, HDV, HTLV I/II, And HIV infection. Bangladesh J. Med. Sci..

[100] Yazdani R., Dadmanesh M., Ghorban K. (2018). First report of the prevalence of human T-lymphotropic virus type 1 (HTLV-1) for hemodialysis patients in Tehran. Arch. Clin. Infect. Dis..

[101] Arjmandi F., Shahriari M., Sadeghi H.M. (2003). A Comparitive study of the prevalence of HTLV–I infection in luekemia/non–hodgkins lymphoma patients, thalassemic patients and blood donors. J. Shahid Sadoughi Univ. Med. Sci..

[102] Moradi A., Mansurian A.R., Ahmadi A.R., Ghaemi E., Kalavi K.H., Marjani A., Sanei Moghaddam E. (2008). Prevalence of HTLV-1 antibody among major thalassemic patients in Gorgan (South East of Caspian Sea). J. Appl. Sci..

[103] ZIAEI M., Zarban A., Malekinejad P., Akhbary H. (2007). Evaluation of HGV viremia prevalence and its co-infection with HBV, HCV, HIV and HTLV-1 in hemophilic patients of Southern Khorassan, Iran. Hepat. Mon..

[104] Rostamzadeh Z., Valizadeh N., Mohammadian M. (2016). Prevalence of seropositivity for human T lymphocytes virus in patients with hereditary bleeding diseases in population of West Azerbaijan. Int. J. Med. Lab..

[105] Ministério da Saúde (BR) (2016). Protocolo de uso da Zidovudina para Tratamento do Adulto com Leucemia/Linfoma Associado ao Vírus HTLV-1.

[106] Bazarbachi A., Plumelle Y., Carlos Ramos J., Tortevoye P., Otrock Z., Taylor G., Gessain A., Harrington W., Panelatti G., Hermine O. (2010). Meta-analysis on the use of zidovudine and interferon-alfa in adult T-cell leukemia/lymphoma showing improved survival in the leukemic subtypes. J. Clin. Oncol. Off. J. Am. Soc. Clin. Oncol..

[107] Kudo H., Fukushima S., Masuguchi S., Sakai K., Jinnin M., Ihn H. (2012). Cutaneous type adult T-cell leukaemia/lymphoma successfully treated with narrowband ultraviolet B phototherapy. Clin. Exp. Dermatol..

[108] Takemori N., Hirai K., Onodera R., Saito N., Yokota K., Kinouchi M., Takahashi H., Iizuka H. (1995). Satisfactory remission achieved by PUVA therapy in a case of crisis-type adult T-cell leukaemia/lymphoma with generalized cutaneous leukaemic cell infiltration. Br. J. Dermatol..

[109] Sawada Y., Shimauchi T., Yamaguchi T., Okura R., Hama-Yamamoto K., Fueki-Yoshioka H., Ohmori S., Yamada S., Yoshizawa M., Hiromasa K. (2013). Combination of skin-directed therapy and oral etoposide for smoldering adult T-cell leukemia/lymphoma with skin involvement. Leuk. Lymphoma.

[110] Takasaki Y., Iwanaga M., Imaizumi Y., Tawara M., Joh T., Kohno T., Yamada Y., Kamihira S., Ikeda S., Miyazaki Y. (2010). Long-term study of indolent adult T-cell leukemia-lymphoma. Blood.

[111] Gill P.S., Harrington W., Kaplan M.H., Ribeiro R.C., Bennett J.M., Liebman H.A., Bernstein-Singer M., Espina B.M., Cabral L., Allen S. (1995). Treatment of adult T-cell leukemia-lymphoma with a combination of interferon alfa and zidovudine. N. Engl. J. Med..

[112] Barbeau B., Hiscott J., Bazarbachi A., Carvalho E., Jones K., Martin F., Matsuoka M., Murphy E.L., Ratner L., Switzer W.M. (2014). Conference highlights of the 16th International Conference on Human Retrovirology: HTLV and related retroviruses, 26–30 June 2013, Montreal, Canada. Retrovirology.

[113] Uozumi K. (2010). Treatment of adult T-cell leukemia. J. Clin. Exp. Hematop..

[114] Tsukasaki K., Utsunomiya A., Fukuda H., Shibata T., Fukushima T., Takatsuka Y., Ikeda S., Masuda M., Nagoshi H., Ueda R. (2007). VCAP-AMP-VECP compared with biweekly CHOP for adult T-cell leukemia-lymphoma: Japan Clinical Oncology Group Study JCOG9801. J. Clin. Oncol. Off. J. Am. Soc. Clin. Oncol..

[115] Di Venuti G., Nawgiri R., Foss F. (2003). Denileukin diftitox and hyper-CVAD in the treatment of human T-cell lymphotropic virus 1-associated acute T-cell leukemia/lymphoma. Clin. Lymphoma.

[116] Teshima T., Akashi K., Shibuya T., Taniguchi S., Okamura T., Harada M., Sumida I., Hanada M., Niho Y. (1990). Central nervous system involvement in adult T-cell leukemia/lymphoma. Cancer.

[117] Hande K.R., Garrow G.C. (1993). Acute tumor lysis syndrome in patients with high-grade non-Hodgkin’s lymphoma. Am. J. Med..

[118] Tsukasaki K., Maeda T., Arimura K., Taguchi J., Fukushima T., Miyazaki Y., Moriuchi Y., Kuriyama K., Yamada Y., Tomonaga M. (1999). Poor outcome of autologous stem cell transplantation for adult T cell leukemia/lymphoma: A case report and review of the literature. Bone Marrow Transplant..

[119] Utsunomiya A., Choi I., Chihara D., Seto M. (2015). Recent advances in the treatment of adult T-cell leukemia-lymphomas. Cancer Sci..

[120] Hishizawa M., Kanda J., Utsunomiya A., Taniguchi S., Eto T., Moriuchi Y., Tanosaki R., Kawano F., Miyazaki Y., Masuda M. (2010). Transplantation of allogeneic hematopoietic stem cells for adult T-cell leukemia: A nationwide retrospective study. Blood.

[121] Ishida T., Utsunomiya A., Iida S., Inagaki H., Takatsuka Y., Kusumoto S., Takeuchi G., Shimizu S., Ito M., Komatsu H. (2003). Clinical significance of CCR4 expression in adult T-cell leukemia/lymphoma: Its close association with skin involvement and unfavorable outcome. Clin. Cancer Res. Off. J. Am. Assoc. Cancer Res..

[122] Sugio T., Kato K., Aoki T., Ohta T., Saito N., Yoshida S., Kawano I., Henzan H., Kadowaki M., Takase K. (2016). Mogamulizumab Treatment Prior to Allogeneic Hematopoietic Stem Cell Transplantation Induces Severe Acute Graft-versus-Host Disease. Biol. Blood Marrow Transplant. J. Am. Soc. Blood Marrow Transplant..

[123] Bai X.T., Moles R., Chaib-Mezrag H., Nicot C. (2015). Small PARP inhibitor PJ-34 induces cell cycle arrest and apoptosis of adult T-cell leukemia cells. J. Hematol. Oncol..

[124] Pujol R.M., Gallardo F. (2021). Cutaneous Lymphomas—Part I: Mycosis Fungoides, Sézary Syndrome, and CD30+ Cutaneous Lymphoproliferative Disorders. Actas Dermo-Sifiliográficas.

[125] Dobos G., Pohrt A., Ram-Wolff C., Lebbé C., Bouaziz J.D., Battistella M., Bagot M., de Masson A. (2020). Epidemiology of Cutaneous T-Cell Lymphomas: A Systematic Review and Meta-Analysis of 16,953 Patients. Cancers.

[126] Scarisbrick J.J., Quaglino P., Prince H.M., Papadavid E., Hodak E., Bagot M., Servitje O., Berti E., Ortiz-Romero P., Stadler R. (2019). The PROCLIPI international registry of early-stage mycosis fungoides identifies substantial diagnostic delay in most patients. Br. J. Dermatol..

[127] Maguire A., Puelles J., Raboisson P., Chavda R., Gabriel S., Thornton S. (2020). Early-stage Mycosis Fungoides: Epidemiology and Prognosis. Acta Derm. Venereol..

[128] Al-Ghamdi H.S. (2020). The prevalence and histological patterns of malignant skin tumors in Albaha, Saudi Arabia: A retrospective study. Med. Sci..

[129] Mufti S.T. (2012). Pattern of skin cancer among Saudi Patients who attended King AbdulAziz University Hospital between Jan 2000 and Dec 2010. J. Saudi Soc. Dermatol. Dermatol. Surg..

[130] Al-Maghrabi J.A., Al-Ghamdi A.S., Elhakeem H.A. (2004). Pattern of skin cancer in Southwestern Saudi Arabia. Saudi Med. J..

[131] Albasri A.M., Borhan W.M. (2018). Histopathological pattern of skin cancer in Western region of Saudi Arabia. An 11 years experience. Saudi Med. J..

[132] Al-Dawsari N.A., Amra N. (2016). Pattern of skin cancer among Saudi patients attending a tertiary care center in Dhahran, Eastern Province of Saudi Arabia. A 20-year retrospective study. Int. J. Dermatol..

[133] Alwunais K.M., Ahmad S. (2016). Pattern of skin cancer at Dammam Medical Complex in Dammam, Saudi Arabia. J. Dermatol. Dermatol. Surg..

[134] Hamodat Mowafak M. (2021). Mycosis Fungoides in UAE. Saudi J. Pathol. Microbiol..

[135] Anadolu R.Y., Birol A., Sanlı H., Erdem C., Türsen Ü. (2005). Mycosis fungoides and Sezary syndrome: Therapeutic approach and outcome in 113 patients. Int. J. Dermatol..

[136] Alsaleh Q.A., Nanda A., Al-Ajmi H., Al-Sabah H., Elkashlan M., Al-Shemmari S., Demierre M.F. (2010). Clinicoepidemiological features of mycosis fungoides in Kuwait, 1991–2006. Int. J. Dermatol..

[137] Naeini F.F., Najafian J., Salehi M., Azimi Z., Rajabi P. (2012). Mycosis Fungoides: Epidemiology in Isfahan, Iran. J. Cosmet. Dermatol. Sci. Appl..

[138] Abdel-Halim M., El-Nabarawy E., El Nemr R., Hassan A.M. (2015). Frequency of hypopigmented mycosis fungoides in Egyptian patients presenting with hypopigmented lesions of the trunk. Am. J. Dermatopathol..

[139] Alojail H.Y., Alshehri H., Kaliyadan F. (2022). Clinical Patterns and Treatment Response of Patients With Mycosis Fungoides a Retrospective Study. Cureus.

[140] AlGhamdi K.M., Arafah M.M., Al-Mubarak L.A., Khachemoune A., Al-Saif F.M. (2012). Profile of mycosis fungoides in 43 Saudi patients. Ann. Saudi Med..

[141] Al-Mufti S. (2013). Morphological Pattern of Mycosis Fungoides Among Saudi Patients Attending King Abdulaziz University Hospital. Turk. Dermatoloji Derg..

[142] Hassab-El-Naby H.M., El-Khalawany M.A. (2013). Hypopigmented mycosis fungoides in Egyptian patients. J. Cutan. Pathol..

[143] El Sayed H., Shalaby S., Abdel-Halim M.R.E., Aboelfadl D.M., Samir N. (2021). Efficacy of doxycycline in the treatment of early stages of mycosis fungoides: A randomized controlled trial. J. Dermatol. Treat..

[144] Binamer Y. (2017). Cutaneous T-cell lymphoma in Saudi Arabia: Retrospective single-center review. Ann. Saudi Med..

[145] Al-Tarawneh A.H. (2018). Clinical and histopathological spectrum of mycosis fungoides. Bahrain Med. Bull..

[146] Haddadin W.J. (2005). Malignant lymphoma in Jordan: A retrospective analysis of 347 cases according to the World Health Organization classification. Ann. Saudi Med..

[147] Tang T., Tay K., Quek R., Tao M., Tan S.Y., Tan L., Lim S.T. (2010). Peripheral T-cell lymphoma: Review and updates of current management strategies. Adv. Hematol..

[148] Schmitz N., Trümper L., Ziepert M., Nickelsen M., Ho A.D., Metzner B., Peter N., Loeffler M., Rosenwald A., Pfreundschuh M. (2010). Treatment and prognosis of mature T-cell and NK-cell lymphoma: An analysis of patients with T-cell lymphoma treated in studies of the German High-Grade Non-Hodgkin Lymphoma Study Group. Blood.

